# Supplementary material for: Evidence supported by Mendelian randomization: impact on inflammatory factors in knee osteoarthritis
Source: Front Med (Lausanne). 2024 May 28;11:1382836. doi: 10.3389/fmed.2024.1382836 (PMC11165061; doi:10.3389/fmed.2024.1382836)
Supplement: Supplementary file 1 [file Data_Sheet_1.ZIP › data/GCST90274821_5e6/821.forest.pdf]

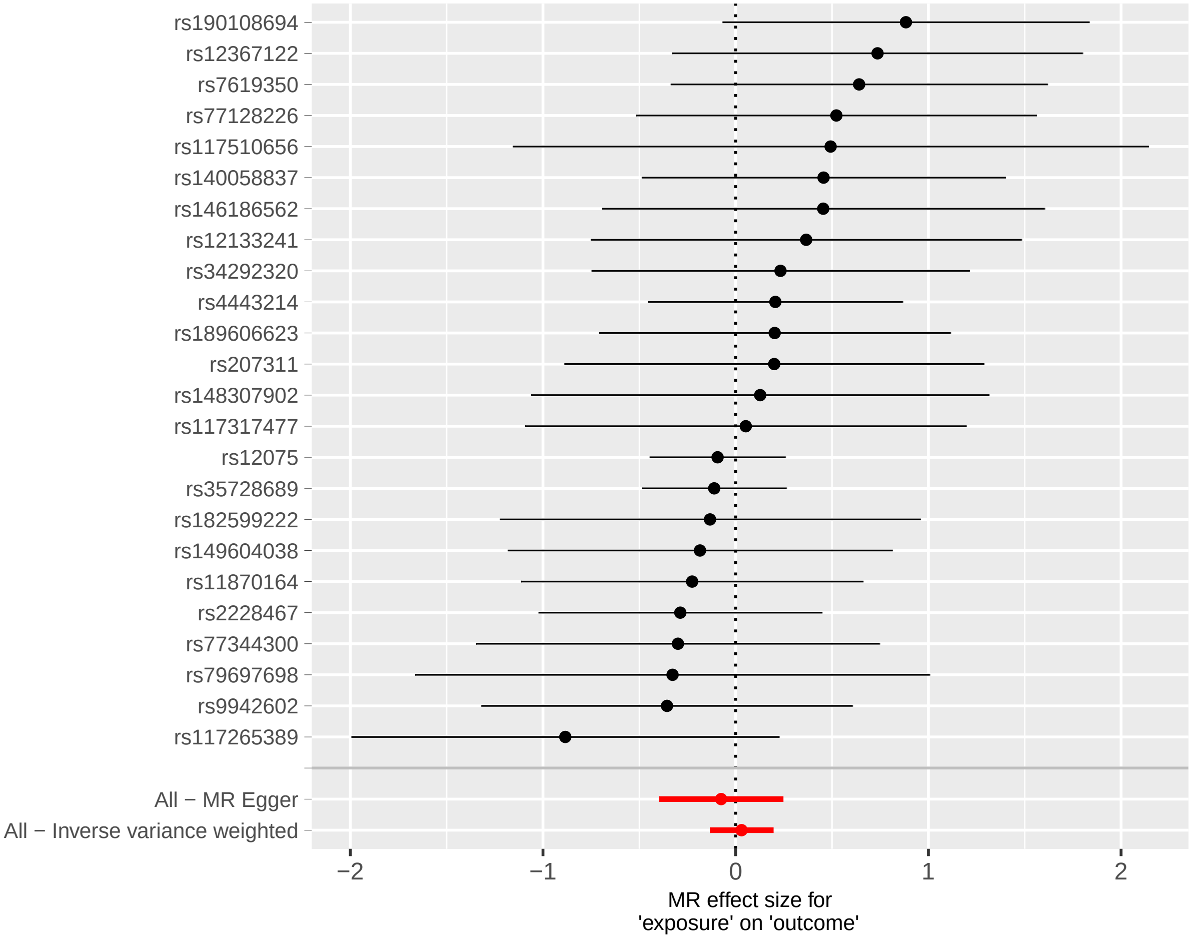

rs190108694
rs12367122
rs7619350
rs77128226
rs117510656
rs140058837
rs146186562
rs12133241
rs34292320
rs4443214
rs189606623
rs207311
rs148307902
rs117317477
rs12075
rs35728689
rs182599222
rs149604038
rs11870164
rs2228467
rs77344300
rs79697698
rs9942602
rs117265389
All − MR Egger
All − Inverse variance weighted
−2
−1
0
1
2
MR effect size for
'exposure' on 'outcome'
